# “SpezPat”- common advance directives versus disease-centred advance directives: a randomised controlled pilot study on the impact on physicians’ understanding of non-small cell lung cancer patients’ end-of-life decisions

**DOI:** 10.1186/s12904-022-01057-5

**Published:** 2022-09-28

**Authors:** Julia Felicitas Leni Koenig, Thomas Asendorf, Alfred Simon, Annalen Bleckmann, Lorenz Truemper, Gerald Wulf, Tobias R. Overbeck

**Affiliations:** 1 Department of Haematology and Medical Oncology, University Medical Centre, Robert-Koch-Str. 40, Goettingen, Germany; 2 Department of Medical Statistics, University Medical Centre, Von-Siebold-Str. 3, Goettingen, Germany; 3 Academy of Ethics in Medicine, Robert-Koch-Str. 40, Goettingen, Germany; 4 Department of Medicine A; Hematology, Oncology and Pneumology, University Hospital Muenster; Albert-Schweitzer-Campus 1, Muenster, Germany

**Keywords:** Advance directive, Palliative care, End-of-life decision making, Prospective pilot study

## Abstract

**Background:**

The advance directive represents patients’ health care choices and fosters patients’ autonomy. Nevertheless, understanding patients’ wishes based on the information provided in advance directives remains a challenge for health care providers. Based on the ethical premises of positive obligation to autonomy, an advanced directive that is disease-centred and details potential problems and complications of the disease should help health care providers correctly understand patients’ wishes. To test this hypothesis, a pilot-study was conducted to investigate whether physicians could make the correct end-of-life decision for their patients when patients used a disease-centred advance directive compared to a common advance directive.

**Material and methods:**

A randomised, controlled, prospective pilot study was designed that included patients with non-small cell lung cancer (NSCLC) stage VI from the Department of Haematology and Medical Oncology, University Medical Centre, Goettingen. Patients were randomised into intervention and control groups. The control group received a common advance directive, and the intervention group received a disease-centred advance directive. Both groups filled out their advance directives and returned them. Subsequently, patients were asked to complete nine medical scenarios with different treatment decisions. For each scenario the patients had to decide whether they wanted to receive treatment on a 5-point Likert scale. Four physicians were given the same scenarios and asked to decide on the treatment according to the patients’ wishes as stated in their advance directives. The answers by patients and physicians were then compared to establish whether physicians had made the correct assumptions.

**Results:**

Recruitment was stopped prior to reaching anticipated sample target. 15 patients with stage IV NSCLC completed the study, 9 patients were randomised into the control group and 6 patients in the intervention group. A total of 135 decisions were evaluated. The concordance between physicians’ and patients’ answers, was 0.83 (95%-CI 0.71–0.91) in the intervention group, compared to 0.60 (95%-CI 0.48–0.70) in the control group, and the difference between the two groups was statistically significant (*p* = 0.005).

**Conclusion:**

This pilot study shows that disease-centred advance directives help physicians understand their NSCLC patients’ wishes more precisely and make treatment choices according to these wishes.

**Trial registration:**

The study is registered at the German Clinical Trial Register (no. DRKS00017580, registration date 27/08/2019).

**Supplementary Information:**

The online version contains supplementary material available at 10.1186/s12904-022-01057-5.

## Introduction

Treating patients with cancer diagnosis remains an important remit in palliative care due to the associated symptom burden and limited life expectancy [1, 2]. Due to the increasing number of treatment options in cancer care and their effects on the patients’ quality of life, it also presents a challenge to advance care planning [1].

Therefore, advance directives as part of advance care planning need to be adapted to this challenge to incorporate the changing dynamics of cancer care.

Autonomy is one of the four principles of bioethics in modern medicine [3]. To foster and preserve autonomy presents a challenge in every physician–patient’s relationship, which is aggravated when patients can no longer make decisions themselves because they lack the capacity to do so. To address this challenge, the concept of advance directives was introduced [3]. The advance directive is a legal document containing patient’s health care choices and wishes to ensure medical treatment according to these wishes, thereby fostering the patient’s autonomy [3]. Nevertheless, the concept of advance directives has been met with insightful criticism on different levels [3–7]. A major concern in the past were problems with implementation [7]. Those were successfully addressed with the introduction of advance care planning. The concept of advance care planning was evaluated in many important scientific studies [1, 8, 9]. Ethical questions remained [10–13]. Among them are questions regarding the nature of understanding, choice and decision making in health care [7, 14]. One of these questions concerning the advance directive as part of advance care planning is how physicians can empower patients to make autonomous choices about end-of-life-decisions. What kind of information do patients need, what important decisions need to be made, how do they relate to the life the patient is currently living and how can these decisions be made in accordance with the patient’s own values? Based on the premises of positive obligation to autonomy, the health care system is obligated to provide patients with the information they need to make autonomous choices [3]. If the premises of positive obligation to autonomy are transferred to the concept of advance directives, the content of the advance directives needs to be adapted. It should be more specific and centre on the likely course and complications of the patient’s disease. A broad document that covers different complications without regards to what is likely to occur as the disease progresses does not fulfill the premise of positive obligation to autonomy.

In conclusion to foster patients’ autonomy it is necessary to adapt the content of the advance directive to focus on the disease that the patient has and its complications.

But patients’ autonomy is only fostered when it is respected as well and acted upon. This is only possible if health care professionals understand the patients’ wishes documented in the advance directive correctly.

In theory, an advanced directive that is disease-centred should foster patients’ autonomy and therefore help health care providers to better understand patients’ wishes. To test this hypothesis, we conducted a pilot study to compare disease-centred advance directives with common advance directives and assessed which one helped health care providers to better understand their patients’ wishes.

## Material and methods

This pilot study was conducted under the premises that a person is capable of making decision in advance for their future self [3–5]. We designed a randomised, controlled, prospective pilot study. The study was conducted at the Department of Haematology and Medical Oncology, University Medical Centre, Goettingen with support of the Academy of Ethics in Medicine and the Department of Medical Statistics, University Medical Centre, Goettingen. A prospective randomised interventional design was used. The study was approved by the local ethics committee (no.14/11/18) and the CONSORT 2010 checklist was used for reporting.

The study aimed to investigate whether a disease-centred advance directive helps physicians understand their patients’ wishes more precisely than a common advance directive.

### Compilation of the disease-centred advance directive

We developed a disease-centred advance directive for patients with non-small cell lung cancer (NSCLC) stage VI. To assess the relevant information that patients with NSCLC stage VI need to be provided with, we made a list of common complications associated with NSCLC stage VI as well as their therapeutic options. The list was derived from standardised consents forms on cancer treatment and its side effects and complications described in oncological textbooks [15, 16] The outline of the disease-centred advance directives was similar to the outline of common advance directives [17]. It started with an introduction, followed by a declaration of validity, a general passage about how to reflect on one’s values, an explanation of all relevant terms used in the advance directive, a section featuring relevant medical scenarios and concluded with the appointment of a health-care proxy. Each medical scenario included a bullet-point list of medical treatment options. Patients could exclude treatments they did not wish to receive by marking them on the bullet-point list. All listed medical scenarios were derived from the list of complications associated with NSCLC stage VI and their therapeutic options.

### Recruitment and intervention

The study included all patients with NSCLC stage VI who were older than 18 years, capable of understanding the study design, the advance directives and giving consent. A sufficient proficiency of the German language was required, as well. Exclusion criteria were a second type of malignancy. As baseline characteristics we documented gender, whether patients had already completed an advance directive prior to the study, whether they had children and whether they considered themselves to be religious. Duration of NSCLC disease was not part of the inclusion criteria. Patients were recruited from January 2019 until December 2020 at the wards and the outpatient clinic of the Department of Haematology and Medical Oncology, University Medical Centre, Goettingen. All cases with NSCLC who are treated at the Department of Haematology and Medical Oncology, University Medical Centre, Goettingen are discussed in an interdisciplinary board of experts who meet once a week. Their recommendations are uploaded to the hospitals database. In order to find eligible patients for that study those recommendations were screened regularly. Eligible patients were contacted in person by the principal investigator at the outpatient clinic or the ward and were asked to participate in the study after its purpose and the aim of the study was explained to them. After giving informed consent, patients were randomised into intervention and control groups (1:1 allocation). The control group received a common advance directive (the advance directive of the Bavarian state ministry of justice), whereas the intervention group received a disease-centred advance directive. Both groups were asked to fill out their advance directives and return them via mail. Once the advance directive was returned, patients were asked to complete a questionnaire.The questionnaire contained nine medical scenarios with different treatment decisions. For each of the nine scenarios the patients had to decide whether they wanted to receive treatment under the nine described circumstances and mark their decision on a 5-point-Likert scale. The nine scenarios included different complications that commonly arise during treatment and progression of NSCLC (see Table 1). As this was a pilot study, all advance directives were only used for study purposes and therefore hypothetical and not part of advance care planning. Patients could receive a copy of their advance directive if they wished to after returning the questionnaires. Patients were therefore asked to complete the documents without professional assistance.

**Table 1:** Content of medical scenarios

Scenario no	Description
**1.1–1.3**	Patient receives ongoing treatment, under which disease is controlled. Treatment is tolerated well, but the patient cannot do household chores alone or leisure activities
**1.1**	Complication: infection and delirium
	Decision: intensive care, yes or no
**1.2**	Complication: infection and delirium
	Decision: resuscitation, yes or no
**1.3**	Complication: infection and delirium
	Decision: antibiotic treatment, yes or no
**2.1–2.3**	Patient receives ongoing treatment, under which disease is not controlled and a new line of treatment is planned. Prior treatment was tolerated well, but the patient cannot do household chores alone or leisure activities
**2.1**	Complication: infection and delirium
	Decision: intensive care, yes or no
**2.2**	Complication: infection and delirium
	Decision: resuscitation, yes or no
**2.3**	Complication: infection and delirium
	Decision: antibiotic treatment, yes or no
**3.1–3.3**	Patient receives ongoing treatment, which is very exhausting for the patient who is bedridden and does not leave the house anymore
**3.1**	Complication: infection and delirium
	Decision: intensive care, yes or no
**3.2**	Complication: infection and delirium
	Decision: resuscitation, yes or no
**3.3**	Complication: infection and delirium
	Decision: antibiotic treatment, yes or no

After all questionnaires were returned via mail, the returned advance directives were distributed among four physicians. The four physicians were given the same scenarios and asked to decide what treatment the patients wanted according to the patients’ wishes as stated in the advance directive. Only physicians who were trained for more than two years and had experience in intensive care were eligible. To ensure an even evaluation, the advanced directives from the intervention and the control group were distributed evenly among physicians, meaning that each of the physicians received the same number of advanced directives from the intervention and the control group respectively patients’ and physicians’ responses were then compared to evaluate whether the physicians had made the correct assumptions.

### Sample size assessment

The required sample size was calculated prior to the trial to confirm a clinically relevant effect. With a sample size of 60 patients (30 per group), assuming a true concordance of 50% between patients and medical doctors in the control group, a significant difference between groups can be observed with a predictive power of 81.9% (two-sided significance level of 5%) if the concordance is at least 62% in the intervention group. Calculations were performed by 1000 simulation runs using generalized linear models (logistic regression) in R Version 4.0.2 [18].

### Data management and statistical analysis

Data collection and management was performed in SecuTrial® via web-based electronic capture report forms. The primary endpoint, i.e., concordance between physician’s and patient’s treatment decisions, was compared between groups using generalized linear mixed models (logistic regression) with random intercept and treatment group as the relevant factor. Concordance rates were reported group-wise with 95%-Confidence Intervals (CI). The pilot study was single-blind. Block randomization with random block length was performed, stratified for the evaluating physicians. Baseline characteristics are reported using descriptive summary values (mean, standard deviation, quartiles, minimum, maximum and range) or through tables and banners for categorical data. All analyses were planned in a statistical analysis plan prior to database locking and were performed in R version 4.0.2.

## Results

**Fig. 1:**
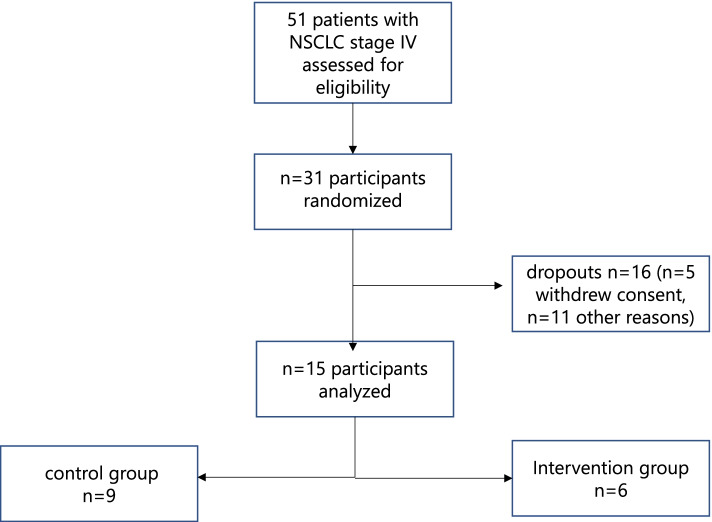
Recruitment flow chart

The study was funded with the starter grant of the Faculty of Medicine, University Medical Centre, Goettingen (Startförderung Forschungsprojekte), which was only available for two years. The study focused on cancer patients with NSCLC stage IV. The study was planned to stop recruitment in 2020. Initially only patients with newly diagnosed NSCLC stage VI were included in the study. As the recruitment rate was very low, an amendment was made to include all patients with NSCLC stage VI in the study. Recruitment was stopped prior to reaching anticipated sample target because the planned sample target could not be reached in the given timeframe of the study and funding was only available until December 2020. 51 eligible patients were contacted (Fig. 1). Of those contacted, 31 patients gave informed consent and were randomized. However, only fifteen completed the study. Of those fifteen participants nine were randomised into the control group and six became part of the intervention group. Baseline characteristics were distributed as presented in Table 2.

**Table 2:** Descriptive data showing absolute numbers or mean and standard deviation (SD), median and minimum/maximum (min/max)

		**Control**	**Intervention**
**Gender**	Male	4	4
	Female	5	2
**Age**	Mean (SD)	67.1 (7.3)	69.5 (8.1)
	Median (Min/Max)	68 (52/75)	71.5 (58/80)
**Do you have children?**	Yes	8	6
	No	1	0
**Are you religious?**	Yes	6	5
	No	2	1
	Missing	1	0
**Do you already have an advance directive?**	Yes	5	4
	No	4	2

**Fig. 2:**
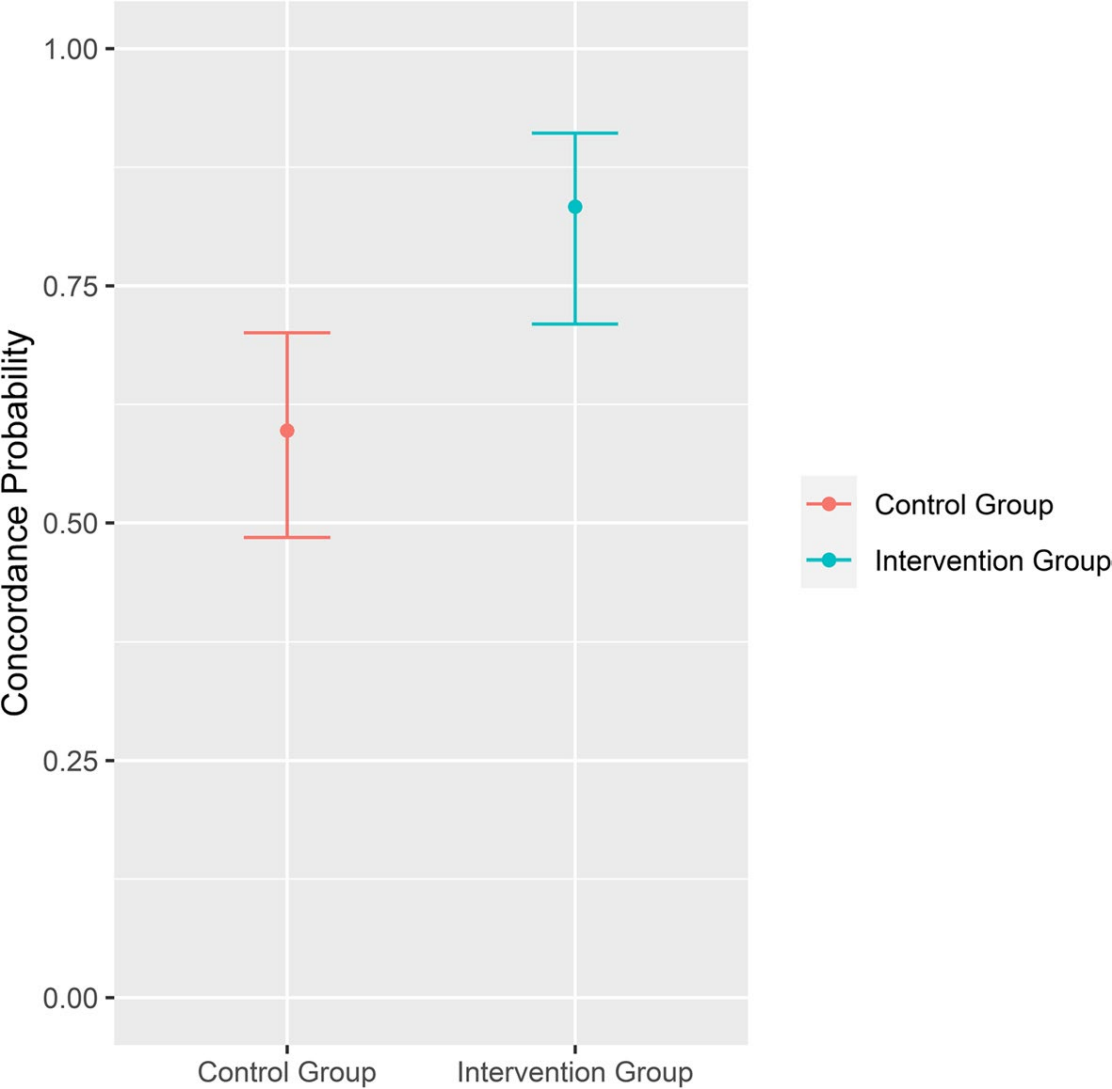
Comparison of concordance probability between control and intervention group

**Fig. 3:**
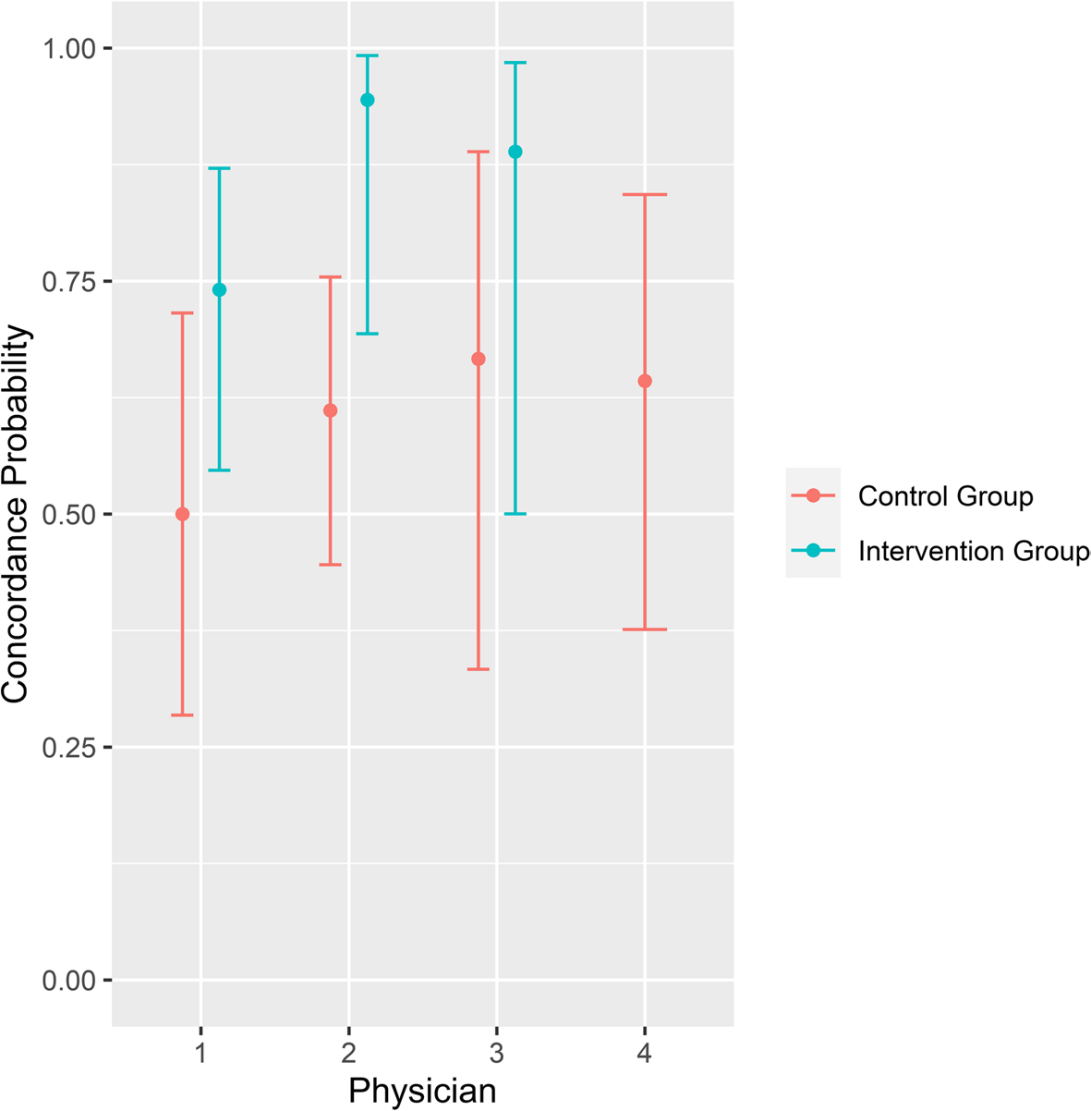
Comparison of concordance probability between the four physicians in intervention and control group; due to the uneven number of participants physician no. 4 exclusively evaluated advance directives from the control group

Since each participant made a treatment decision for all nine medical scenarios, a total of 135 decisions were evaluated. The primary endpoint, concordance of physicians’ and patient’s answers, was measured to be 0.83 (95%-CI 0.71–0.91) in the intervention group, compared to 0.60 (95%-CI 0.48–0.70) in the control group (Fig. 2). The difference was shown to be statistically significant (*p* = 0.005). Sensitivity analysis to assess the influence of the physicians’ rating and specific scenarios could not be performed due to low sample sizes. Four physicians (two from the Department of Haematology and Medical Oncology and two from the Department of Cardiology and Pneumology) performed the evaluation. Even distribution of control and intervention advanced directives was not possible due to the uneven number of participants. Concordance with the patients’ wishes was higher in the intervention group for each physician, except for one who evaluated only advanced directives from the control group. Descriptive analysis showed that concordance probabilities only differed between intervention group and control group and did not reveal any evidence of unequal rates between physicians (Fig. 3).

**Fig. 4:**
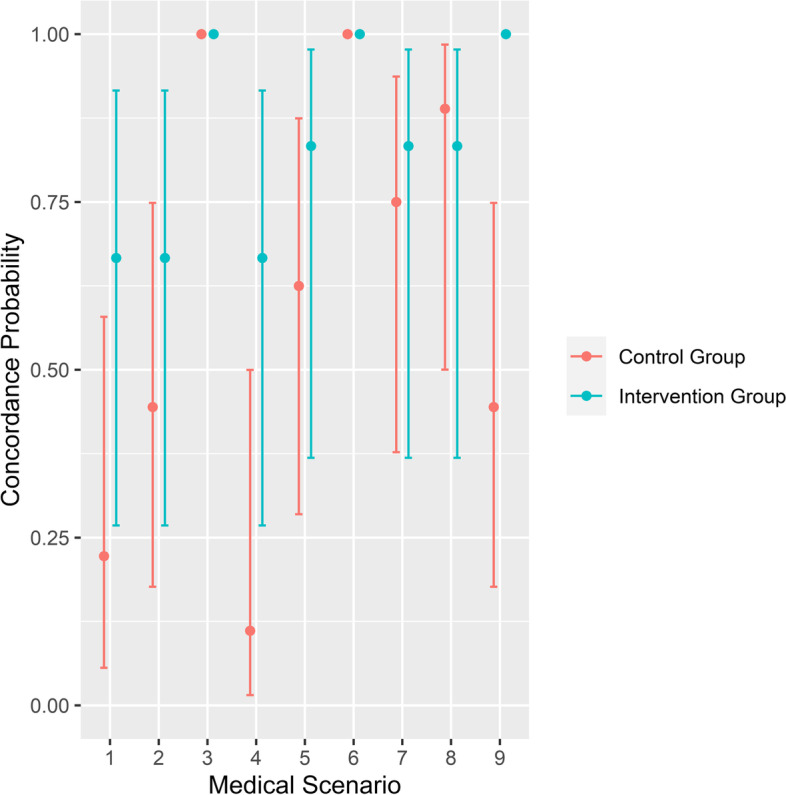
Comparison of concordance probability in intervention and control group between the different medical scenarios

When comparing the different scenarios, concordance probabilities were higher in the intervention group than in the control group in all scenarios except 3, 6 and 8 (Fig. 4). In scenarios 3 and 6, control and intervention both had a concordance probability of 1, suggesting that these scenarios are less difficult to decide on. Scenario 9 showed a concordance rate of 1 in the intervention group. In scenario 8, the control group had a concordance probability of 0.88 and intervention group had a concordance probability of 0.83 (Fig. 4). Therefore, the physicians had more difficulty in interpreting the patients’ disease-centred advance directive in this scenario.

## Discussion

### Limitations

The study presented here has some limitations. It included only patients with NSCLC and no other type or stage of cancer. The sample size is low, with only fifteen patients completing the pilot study. Initially, a much larger sample target was planned, which could not be reach because of the study’s timeframe and the availability of funding. Acquisition was therefore stopped prior to reaching anticipated sample target. Of 51 eligible patients only 31 wanted to participate in the study and of those 31 patients only fifteen completed it. This might be due to the data collection method. Patients received and returned the documents via email. Mail surveys in general have response rates of around 60% [19]. Another reason could be the negative emotional reactions that was related to considering end-of-life and end-of-life decisions. Negative emotional reactions were a concern when the study was designed. Therefore, it was part of the informed consent process to explain to the patients that participation in this study could trigger anxiety and depression and that the advance directive that was completed as part of the pilot study was not binding and would only be used for study purposes. Furthermore, the Department of Psychooncology, University Medical Centre Goettingen was informed about the study and their contact details were added to the informed consent form. After consultation with the local ethics commission, we did not assess the reasons for drop-out so as to refrain from triggering negative emotional reactions.

Another limitation is that the duration of history of disease was different between the participants. Some were newly diagnosed with stage IV NSCLC and had just started treatment and others had received treatment for years. The initial study design only included patients that had been newly diagnosed with NSCLC stage IV and had just started treatment. As recruitment rates were very low, the inclusion criteria were adapted to include all patients with NSCLC stage IV. The initial concern was that a bias would be introduced as participants with a long history of disease and treatment might be more precise in their advance directives, as they are more familiar with complications and course of disease.

None of the four physicians who were asked to evaluate what treatment the patients wanted according to the patients’ wishes as stated in the advance directive were trained in advanced care planning or advanced directives. Two of them were specialised in internal medicine and two were training to specialise in internal medicine. They all had experience with advance directives in acute care and the problems acute care providers face when confronted with advance directives [20].

The disease-centered advance directive was tested only against the advance directive of the Bavarian state ministry of justice. Other advance directives like the Physician Orders for Life-Sustaining Treatment (POLST)-form [21] were not tested. Therefore, results are limited to the difference between these two advance directives.

The advance directive of the Bavarian state ministry of justice was chosen because it is recommended by a German government institute and was designed to foster patients’ autonomy. Therefore, it contains explanations of medical terms and legal aspect for a layperson while describing possible scenarios.

Underlying premise of this pilot study is the concept of positive obligation to autonomy which requires that the health care system provides patients with the information they need to make autonomous choices. The authors are aware that there is a concept of negative obligation to autonomy but reject it. The POLST-form is limited to a few decisions without any context. The authors perceive a patient’s history of disease and his or her decisions as influenced by many more factors and do not believe that end-of-life decision making is only limited to DNR/DNI-decision but more complex and depending on the situation the patient is in right now.

Furthermore, the questionnaires that included the nine scenarios were developed especially for the purpose of this study and are therefore also pilot scenarios that need to be revised and validated in further studies. To put the results into a broader perspective the results and methods need to be validated in larger studies following this pilot study.

### Discussion of results

The aim of the pilot study was to assess the advance directive in patients with NSCLC which is only a part of advance care planning and not advance care planning as a whole. This data suggests that disease-centred advance directives might help physicians understand their NSCLC patients’ wishes more precisely and make treatment choices according to their wishes. Even though the sample size was not large enough to perform a sensitivity analysis, a descriptive analysis showed that when physicians made treatment decisions with the help of the disease-centred advance directive, decisions were more likely to be according to the patients’ wishes than when they made decisions with the help of the common advance directive.

These results need to be confirmed with a larger sample size, but results suggest that there was no underlying confounder. A disease-centred advance directive might indeed help physicians better understand their NSCLC patients’ wishes.

Except for scenario 3, 6 and 8, results show that in most scenarios, physicians understood the patients’ treatment decisions better when a disease-centred advance directive was used. Scenario 3 and 6 showed no difference between the treatment decision of patients and physicians in control or intervention group. In scenario 8 there was only a minor difference. Therefore, scenarios 3, 6 and 8 do not provide the statistical discriminatory power and these scenarios should be eliminated or altered in a larger study. From a medical care point of view, it is interesting that both scenarios 3 and 6 dealt with the question of receiving antibiotic treatment while the disease remains stable (scenario 3) or progresses (scenario 6). Almost all patients opted for treatment in these scenarios, except a few who were indecisive. Scenario 8 described reduced capability to participate in daily life (severe fatigue) due to the side effects of treatment and asked if participants wanted to be resuscitated in the case of a cardiac arrest. Most participants decided against treatment.

This data suggests that wishes seemed to be communicated well in these instances and independently of which type of advance directives are used.

All nine scenarios that were used in the questionnaire were designed for the purpose of this study as there were no other test scenarios available. Therefore, the content of the scenarios and the phrases used to describe them need to be discussed, adapted and validated in further studies with lager sample sizes.

### Integration into current research

With the implementation of advance care planning, drafting and completing advance directives have become a small part in fostering patients’ autonomy regarding end of life decisions [2, 22–25]. Advance care planning aims to transform the act of writing down one’s treatment wishes into a continuous process that includes structures and procedures to keep conversation about treatment wishes going. The process of advance care planning ensures that changes in patients’ decisions are acknowledged and added to existing advance directives. Furthermore, advance care planning raises general awareness on end-of-life decision making and its importance for patients’ autonomy [26]. Even though the advance directive is only a small piece of advance care planning, it still plays an important role. Studies suggest that advance directives may help to reduce overtreatment and hospital admissions at the end-of-life stage [8, 27, 28]. Still, it remains unclear whether this also means that treatment is in concordance with the patients’ wishes. Most studies use ICU or hospital admission as end points [8] without comparing them to the instructions given in the advance directive. Even though it can be assumed that most patients who possess an advance directive drafted it because they do not want life-prolonging treatment, that is not necessarily true. Up to this point, only a few studies have evaluated whether advance directives actually help health care providers understand the patients’ wishes [29]. Therefore, there is little data on how well health care providers understand patients’ treatment preferences and choices after reading their advance directive. Still, it remains an important question when it comes to end-of-life decision making. Especially when it is not possible to ask patients about their preferred treatment anymore and since there are many types of advance directives that differ greatly in their content and their approach on preserving the patients’ autonomy [30–33]. And yet, there is no established validation process or outcome measurement that shows that the advance directive indeed reflects the patients’ wishes and values and is correctly understood by health care providers.

Data from the acute care setting shows that advance directives remain an important tool to assess patients’ wishes and foster their autonomy in acute care [20, 34, 35]. Unfortunately, acute care providers often describe them as unclear, not applicable to the situation and unhelpful [20]. This underlines the importance of implementing and validating advance directives that help health care providers to understand patients’ treatment wishes. In this study we showed that an advance directive that is disease-centred has the potential to be more helpful.

## Conclusions

Advance directive still play an important role in end-of-life decision making. In this pilot study, we showed that a disease-centred advance directive might help health care providers to understand NSCLC patients’ wishes more precisely. Due to the low sample size, the results need to be validated in a larger prospective study.

## Supplementary Information

**Additional file 1. **Disease-centered advance directive for patientswith non-small cell lung cancer.

**Additional file 2. **Document 2 disease scenarios.

## Data Availability

The datasets used and analysed for this study are not publicly available as they are private in nature and refer to patients’ health. They are, however available upon reasonable request. Please contact the corresponding author (JK).
