# Supplementary material for: “SpezPat”- common advance directives versus disease-centred advance directives: a randomised controlled pilot study on the impact on physicians’ understanding of non-small cell lung cancer patients’ end-of-life decisions
Source: BMC Palliat Care. 2022 Sep 28;21:167. doi: 10.1186/s12904-022-01057-5 (PMC9516789; doi:10.1186/s12904-022-01057-5)
Supplement: Supplementary file 1 — Additional file 1. Disease-centered advance directive for patientswith non-small cell lung cancer. [file 12904_2022_1057_MOESM1_ESM.pdf]

Disease-centered advance directive for patients with non-small cell lung cancer

## Introduction

Illness changes a person's life. Questions and worries that previously played only a minor role or no role at all suddenly come to the fore. For many sick people, this includes worries about the future. It is associated with a variety of wishes. One of these wishes is often to be able to help shape the course of the illness and the treatment and to ensure that one's own values are reflected in it. The advance directive is one way for patients to be able to do this. In the advance directive, a patient can decide on treatment measures and therapies according to his/her own values and put this in writing in case he/she is no longer able to express his/her opinion on these treatments and therapies.

This advance directive was developed specifically for patients with non-small cell lung cancer. This means that special attention has been paid to situations that arise in the context of this disease.

The questions and decisions addressed in an advance directive are not easy questions or decisions. Illness and its consequences can be frightening and saddening. The questions and decisions raised by an advance directive are very personal questions. They contain wishes and values about the very personal idea of a good life. The advance directive helps to realize this idea as it contains decisions for a stage of life in which the patient can no longer decide for him/herself. In this way, the patient's own idea of a good life can also be realised.

Since the idea of a good life can change with the experiences one has in life, it is sensible and important to review the advance directive again and again and to check whether what was documented still agrees with one's own wishes and values.

There are different decision-making situations in medicine, that the advance directive can cover. Since the advance directive only comes into effect when a patient is no longer able to decide for him/herself, it is important to know that there are different types situation in which incapacity occurs.

Incapacity can arise in which the ability to make decisions suddenly no longer exists or in which this is a gradual process. The decisions made in these situations differ not only in the speed with which they have to be made, but also in the fact that the further course of the disease can be predicted with very different accuracy. Typical situations for sudden inability to make decisions are cardiovascular arrest or sudden respiratory failure. Here, quick decisions have to be made and long explanations and conversations are out of place. At the same time, a doctor cannot predict whether the patient will be able to go about his or her daily life again after, for example, resuscitation, as he or she did before the cardiovascular arrest. The questions one has to ask oneself about one's own wishes and values about these situations differ considerably.

The advance directive is not a binding and non-amendable document for the patient and can be changed, destroyed or revoked at any time.

For the reasons mentioned above, it is helpful to share the contents of the advance directive with people close to you. Many decisions and issues addressed in the advance directive can emotionally affect those close to you. Being open about the advance directive and the issues it addresses can reduce anxiety and worry in an emergency. In this way, these people know

about the advance directive and can help to ensure that the advance directive is available and that the wishes in the advance directive are followed.
It is important that the advance directive is accessible and not locked away in a locker or drawer. It should be brought to every inpatient hospital stay and there should be a copy left with the family doctor.

This advance directive is divided into different parts. The first section is for legal validity and confirms the patient's capacity to consent. The second section is intended to help the patient work out his/her own values and wishes. This section contains questions about wishes, values, religious beliefs and attitudes to life, which should help to better describe one's own beliefs. In the third section, some medical terms are explained in more detail for better understanding. In the fourth section some possible medical decision-making situations are listed for which a decision can be made in each case. In the following fifth section, a proxy can be nominated. The sixth section is an emergency sheet that contains clear decisions for emergency situations. Personal additions and comments can be deposited in the seventh section.

## Validity of the order

In the event that I

---

Born on:

---

residing in:

---

I am no longer able express my wishes and will, I make the following orders:

## Determining one's own values

This section of the advance directive is intended to encourage you to think about your own wishes and values:

- What keeps you alive and gives you joy? What is valuable in your life?
- Under what circumstances do you want to live long? Does the intensity of life mean more to you than the duration? Has the disease changed your beliefs about what makes your life valuable?
- Under what circumstances is it not a goal for you to expand the duration of your life? What role does the ability to fully participate in social life play for you?
- When you think of dying; what comes to your mind?
- If you were told now that you would fall asleep peacefully tonight and not wake up tomorrow - what would that mean for you?
- What does it mean for you to still be alive?

- How have you dealt with painful experiences in your life so far? Are you afraid of being a burden to other people or can you also let others help you? How have you and those close to you dealt with the disease? How have you and those close to you dealt with the side effects of the treatment?
- What role does religion play in your life? And what role does it play in your belief beyond death?
- How do other people's disabilities affect you? Do mental disabilities affect you differently than physical disabilities? What thoughts come to you when you see patients with a similar condition? What feelings and thoughts does it trigger in you?
- How has cancer changed your life? Have other things in your life suddenly gained or lost importance?

Consider these questions, take your time. Use the questions as food for thought for the medical decision-making situations presented in the following sections.

## Explanations

Incapacity to consent: This refers to physical conditions in which a person is unable to give consent. There are many possible reasons for this, e.g. confusion due to illness, artificial coma due to ventilation therapy, mental deficiency or brain degeneration. These examples already show that these physical states sometimes only last for a short time and are not reversable. Therefore, some people are incapable of consenting only for a short period of time and some people are incapable of consenting for the rest of their lives. An advance directive is only needed when a person is no longer capable of giving consent and is also only valid for as long as he or she is incapable of giving consent. Incapacity to consent does not have to occur suddenly, but can set in gradually, which sometimes makes it difficult to decide whether someone is capable of consenting or not.

Intensive care: Intensive care uses measures that support the respiratory and cardiovascular functions of a person. Intensive care measures are used for patients who can no longer maintain their circulation or respiratory functions. These patients are life-threateningly ill. Intensive care measures include: ventilation and circulatory support. In order to monitor these intensive care measures, the patient is connected to a surveillance monitor.

Cardiac arrest and resuscitation: Cardiovascular arrest is a situation in which the blood circulation stops and therefore all organs can no longer be supplied with oxygen. Often the cause is cardiac arrest. If cardiac arrest occurs, a person is dead. A person can be resuscitated by cardiac massage and artificial circulatory support. The brain and the heart can only survive a few minutes without oxygen, otherwise great damage will occur. To prevent death, resuscitation must be started immediately. If no resuscitation measures are taken, the person is dead.

Artificial ventilation: artificial ventilation is given when a person is so ill that he or she can no longer adequately maintain their own respiratory function. Artificial ventilation requires an artificial coma so that the patient can be connected to a ventilator via a plastic tube. The artificial coma is induced by drugs that are continuously administered to the patient through the blood system. To monitor the artificial ventilation, a patient is connected to a monitoring system.

Circulatory support: circulatory support is the continuous supply of medication to maintain circulation. Without these drugs, the patient's heart would stop and he or she would die. Drug-induced circulatory support does not always require an artificial coma. To monitor circulatory support, a patient is connected to a monitoring system.

Chemotherapy, immunotherapy, antibody therapy and side effects: Chemotherapy, immunotherapy and antibody therapy are different forms of treatment against cancer. They not only work differently, but also have different side effects. Chemotherapy often has more side effects than immunotherapy or antibody therapy. On the other hand, the side effects of immunotherapy and antibody therapy, if they occur, can also be very severe. There are many different types of chemotherapy, immunotherapy and antibody therapy. How a patient will respond to the therapy and how well he or she will tolerate it cannot be predicted in advance. There are also treatment options for the side effects and some can be prevented by supportive therapy.

## Medical decision-making situations

Artificial ventilation

Intubation and subsequent artificial ventilation is out of the question for me when

- My disease is far advanced and has progressed very rapidly in the last few weeks and therefore the disease is medically assessed to have very little chance of being controlled.
- My well-being is limited treatment and the disease that I can participate less and less in daily life because I sleep a lot and am physically very weak.
- I am already in all probability in the immediate process of dying.
- I have already received a variety of treatment and the cancer is still there.
- The chances that artificial ventilation will restore my physical abilities and my physical condition to the state they were before artificial ventilation are very small.
- Prior to the artificial ventilation, it can already be assumed on the basis of my physical condition that the artificial ventilation is not a temporary condition and the chances that I will ever be able to breathe again without artificial ventilation are very small.
- The disease progression is in such a way that it can only be controlled with a very small chance and my time of death is most likely only delayed.
- I will be bedridden and dependent on help afterwards.
- I am increasingly dependent on help due to therapy and the illness and I can no longer live my everyday life independently.
- There is a probability that I already have brain damage.
- I would like to leave the decision as to whether artificial ventilation should be carried out to the attending physician.
- I generally reject artificial ventilation.

Circulatory support
Circulatory support should be dispensed with if
- My disease is far advanced and has progressed very rapidly in the last few weeks and therefore the disease is medically assessed to have very little chance of being controlled.
- My well-being is limited treatment and the disease that I can participate less and less in daily life because I sleep a lot and am physically very weak.
- I am already in all probability in the immediate process of dying.
- I have already received a variety of treatment and the cancer is still there.
- The chances that drug-assisted circulatory support will restore my physical abilities and condition to the way they were before drug-assisted circulatory support are very low.
- Prior to the circulatory support, it can already be assumed on the basis of my physical condition that the circulatory support is not a temporary condition and that the chances of me ever being able to live without circulatory support again are very small.
- The disease progression is in such a way that it can only be controlled with a very small chance and my time of death is most likely only delayed.
- I will be bedridden and dependent on help afterwards.
- I am increasingly dependent on help due to therapy and the illness and I can no longer live my everyday life independently.
- There is a probability that I already have brain damage.
- I would like to leave the decision as to whether circulatory support should be carried out to the attending physician.
- I generally reject circulatory support.

Reanimation and resuscitation
Resuscitation should not be performed if
- My disease is far advanced and has progressed very rapidly in the last few weeks and therefore the disease is medically assessed to have very little chance of being controlled.
- My well-being is limited treatment and the disease that I can participate less and less in daily life because I sleep a lot and am physically very weak.
- I am already in all probability in the immediate process of dying.
- I have already received a variety of treatment and the cancer is still there.
- The chances that intensive care treatment after resuscitation will restore my physical abilities and my physical condition before resuscitation are very low.
- Before the resuscitation, it can already be assumed on the basis of my physical condition that the intensive care treatment after the resuscitation is not a temporary condition and that the chances of me ever being able to live again without intensive care treatment are very low.
- The disease progression is in such a way that it can only be controlled with a very small chance and my time of death is most likely only delayed.
- I will be bedridden and dependent on help afterwards.
- I am increasingly dependent on help due to therapy and the illness and I can no longer live my everyday life independently.
- There is a probability that I already have brain damage.
- I would like to leave the decision as to whether resuscitation should be carried out to the attending physician.
- I am generally opposed to resuscitation.

Dialysis

Dialysis should not be performed if

- My disease is far advanced and has progressed very rapidly in the last few weeks and therefore the disease is medically assessed to have very little chance of being controlled.
- My well-being is limited treatment and the disease that I can participate less and less in daily life because I sleep a lot and am physically very weak.
- I am already in all probability in the immediate process of dying.
- I have already received a variety of treatment and the cancer is still there.
- The chances that dialysis will preserve my physical abilities and condition are very low.
- If, due to my physical condition, it can be assumed that dialysis is not a temporary condition and that the chances of me ever being able to live without dialysis again are very low.
- The disease progression is in such a way that it can only be controlled with a very small chance and my time of death is most likely only delayed.
- I will be bedridden and dependent on help afterwards.
- I am increasingly dependent on help due to therapy and the illness and I can no longer live my everyday life independently.
- There is a probability that I already have brain damage.
- I would like to leave the decision as to whether dialysis should be carried out to the attending physician.
- I generally refuse dialysis.

Chemotherapy

I refuse further chemotherapy if

- My disease is far advanced and has progressed very rapidly in the last few weeks and therefore the disease is medically assessed to have very little chance of being controlled.
- My well-being is limited treatment and the disease that I can participate less and less in daily life because I sleep a lot and am physically very weak.
- I am already in all probability in the immediate process of dying.
- I have already received a variety of treatment and the cancer is still there.
- The chances that chemotherapy will improve my physical condition in the long term are very low.
- The disease progression is in such a way that it can only be controlled with a very small chance and my time of death is most likely only delayed.
- I am increasingly dependent on help due to therapy and the illness and I can no longer live my everyday life independently.
- There is a probability that I already have brain damage.
- I would like to leave the decision as to whether chemotherapy should be carried out to the the attending physician.

Immunotherapy:

I refuse further immunotherapy if

- My disease is far advanced and has progressed very rapidly in the last few weeks and therefore the disease is medically assessed to have very little chance of being controlled.
- My well-being is limited treatment and the disease that I can participate less and less in daily life because I sleep a lot and am physically very weak.
- I am already in all probability in the immediate process of dying.
- I have already received a variety of treatment and the cancer is still there.
- The chances that immunotherapy will improve my physical condition in the long term are very low.
- The disease progression is in such a way that it can only be controlled with a very small chance and my time of death is most likely only delayed.
- I am increasingly dependent on help due to therapy and the illness and I can no longer live my everyday life independently.
- There is a probability that I already have brain damage.
- I would like to leave the decision as to whether immunotherapy should be carried out to the attending physician.

Antibody therapy:
I refuse further antibody therapy if

- My disease is far advanced and has progressed very rapidly in the last few weeks and therefore the disease is medically assessed to have very little chance of being controlled.
- My well-being is limited treatment and the disease that I can participate less and less in daily life because I sleep a lot and am physically very weak.
- I am already in all probability in the immediate process of dying.
- I have already received a variety of treatment and the cancer is still there.
- The chances that the antibody therapy will improve my physical condition in the long term are very low.
- The disease progression is in such a way that it can only be controlled with a very small chance and my time of death is most likely only delayed.
- I am increasingly dependent on help due to therapy and the illness and I can no longer live my everyday life independently.
- There is a probability that I already have brain damage.
- I would like to leave the decision as to whether an antibody therapy should be carried out to the attending physician.

Surgery
I refuse surgery of any kind if

- My disease is far advanced and has progressed very rapidly in the last few weeks and therefore the disease is medically assessed to have very little chance of being controlled.
- My well-being is limited treatment and the disease that I can participate less and less in daily life because I sleep a lot and am physically very weak.
- I am already in all probability in the immediate process of dying.
- I have already received a variety of treatment and the cancer is still there.
- The chances that the surgery will preserve my physical abilities and condition are very low.

- The disease progression is in such a way that it can only be controlled with a very small chance and my time of death is most likely only delayed.
- I will be bedridden and dependent on help afterwards.
- I am increasingly dependent on help due to therapy and the illness and I can no longer live my everyday life independently.
- There is a probability that I already have brain damage.
- I would like to leave the decision as to whether an operation should be carried out to the attending doctor.
- I generally reject any kind of surgery.

Antibiotic therapy:

I refuse therapy with antibiotics if

- My disease is far advanced and has progressed very rapidly in the last few weeks and therefore the disease is medically assessed to have very little chance of being controlled.
- My well-being is limited treatment and the disease that I can participate less and less in daily life because I sleep a lot and am physically very weak.
- I am already in all probability in the immediate process of dying.
- I have already received a variety of treatment and the cancer is still there.
- The chances that antibiotic therapy will preserve my physical abilities and condition are very slim.
- The disease progression is in such a way that it can only be controlled with a very small chance and my time of death is most likely only delayed.
- I am increasingly dependent on help due to therapy and the illness and I can no longer live my everyday life independently.
- There is a probability that I already have brain damage.
- I would like to leave the decision as to whether an antibiotic therapy should be carried out to the attending physician.

## Surrogate

My relative and other person of trust in accordance with § 1901b Para. 2 of the German Civil Code (BGB) is:

Name, first name, date of birth:

______________________________________________

Address, telephone, fax, e-mail:

______________________________________________

The following person(s) should not be consulted:

Name, first name, date of birth:

______________________________________________

Address, telephone, fax, e-mail:

______________________________________________

## Personal amendments and comments

My personal additions to the advance directive
(current life and illness situation, additional illness conditions with the respective treatment or non-treatment wishes, basic considerations on living and dying).

Name, first name:

Address:

Place, date Signature:
