# Supplementary material for: “SpezPat”- common advance directives versus disease-centred advance directives: a randomised controlled pilot study on the impact on physicians’ understanding of non-small cell lung cancer patients’ end-of-life decisions
Source: BMC Palliat Care. 2022 Sep 28;21:167. doi: 10.1186/s12904-022-01057-5 (PMC9516789; doi:10.1186/s12904-022-01057-5)
Supplement: Supplementary file 2 — Additional file 2. Document 2 disease scenarios. [file 12904_2022_1057_MOESM2_ESM.pdf]

Document 2
Disease scenarios

Zus.-ID:________________ Date: _________

The following situations <u>may or may not</u> occur during the course of your disease. We ask you to consider how you would decide.

<u>Imagine that</u> your physical condition has changed due to therapy and illness. You are not able to do your household chores alone without help or to go on leisure activities. You tolerate the treatment you are currently receiving well. You spend about half a day a week in the outpatient clinic. <u>Under the current therapy, your tumour is stable and shows no changes</u>.
Now an infection occurs. Due to this infection, you develop a delirium (you are confused and disoriented).

Scenario P1.1

Your cardiovascular system is extremely stressed, so that intensive medical care is necessary. Intensive medical care includes artificial coma with ventilation and circulatory support.
Your body is already weakened by the treatment and the tumour, so there is a possibility that you will die despite the intensive care treatment. Your hospital stay will last several weeks and it is very likely that you will be bedridden and dependent on help in the long term.
<u>Do you want intensive care treatment in this situation?</u>

○ not wanted	○ probably not wanted	○ indeterminate	○ Probably wanted	○ wanted

Scenario P1.2

A cardiovascular arrest occurs. If the cardiovascular system can be restored by resuscitation measures, intensive care treatment with artificial coma, ventilation and circulatory support follows. Survival after resuscitation depends on the cause of the cardiovascular arrest and whether damage has occurred in the brain tissue. In the worst case, damage has occurred in the brain tissue and you would no longer be able to communicate with your environment and would be completely dependent on help and bedridden. In the best case, no permanent damage occured and you can be discharged home after a long stay in hospital.
<u>Do you want resuscitation measures in this situation?</u>

○ not wanted	○ probably not wanted	○ indeterminate	○ Probably wanted	○ wanted

Scenario P1.3

You already received several therapies which have only stopped the tumour growth for a certain time.
The infection should now be treated with antibiotics, otherwise it is possible that you will die. With the antibiotics, there is a high probability that the infection can be treated

and that you will be very weak after several weeks in hospital and have little physical strength for everyday and leisure activities.
In this case, do you want antibiotic treatment?

○ not wanted	○ probably not wanted	○ indeterminate	○ Probably wanted	○ wanted

Imagine that your physical condition has changed due to therapy and illness. You are not able to do your household chores alone without help or to go on leisure activities. You tolerated the therapy you were currently receiving well. You are in the day clinic once a week for about half a day. Your tumour has continued to grow under the current therapy and you are to receive a new therapy.
Now an infection occurs as part of your illness. Due to this infection, you develop a delirium (you are confused and disoriented).

Scenario P2.1

Your cardiovascular system is extremely stressed, so that intensive medical care is necessary. Intensive care treatment includes an artificial coma with ventilation and circulatory support.
Your body is already weakened by the therapy and the tumour, so there is a possibility that you will die despite the intensive care treatment. Your hospital stay will last several weeks and you will most likely be discharged bedridden and dependent on assistance.
Do you want intensive care treatment in this situation?

○ not wanted	○ probably not wanted	○ indeterminate	○ Probably wanted	○ wanted

Scenario P2.2

A cardiovascular arrest occurs. If the cardiovascular system can be restored by resuscitation measures, intensive care treatment with artificial coma, ventilation and circulatory support follows. Survival after resuscitation depends on the cause of the cardiovascular arrest and whether damage has occurred in the brain tissue. In the worst case, damage has occurred in the brain tissue and you would no longer be able to communicate with your environment and would be completely dependent on help and bedridden. In the best case, no permanent damage occured and you can be discharged home after a long stay in hospital.
Do you want resuscitation measures in this situation?

○ not wanted	○ probably not wanted	○ indeterminate	○ Probably wanted	○ wanted

Scenario P2.3

You have already received several therapies which have only stopped the tumour for a certain time. The infection should now be treated with antibiotics, otherwise it is possible that you will die. With the antibiotics, there is a high probability that the infection can be treated and that you will be very weak after several weeks in hospital and have little physical strength for everyday and leisure activities.
In this case, do you want antibiotic treatment?

○ not wanted	○ probably not wanted	○ indeterminate	○ Probably wanted	○ wanted

<u>Imagine that</u> your physical condition has changed due to therapy and illness. <u>The treatment is very exhausting for you, so you spend most of your time in bed and sleep a lot.</u> When you are awake, you cannot participate in household chores, you are dependent on help for many everyday activities and <u>you no longer leave the house</u>.
As part of your illness, you now develop an infection. Due to this infection, you develop a delirium (you are confused and disoriented).

Scenario P3.1

Your cardiovascular system is extremely stressed, so that intensive medical care becomes necessary. Intensive care treatment includes an artificial coma with ventilation and medicinal circulatory support.
Your body is already weakened by the therapy and the tumour, so there is a possibility that you will die despite the intensive medical treatment. Your hospital stay will last several weeks and you will most likely be discharged bedridden and dependent on assistance.
<u>Do you want intensive care treatment in this situation?</u>

○ not wanted	○ probably not wanted	○ indeterminate	○ Probably wanted	○ wanted

Scenario P3.2

Cardiovascular arrest occurs. If the cardiovascular system can be restored by resuscitation measures, intensive medical treatment with artificial coma, ventilation and circulatory support follows. Survival after resuscitation depends on the cause of the cardiovascular arrest and whether there is damage to the brain tissue. In the worst case, damage has occurred in the brain tissue and you would no longer be able to communicate with your environment and would be completely dependent on help and bedridden. In the best case, no permanent damage occured and you can be discharged home after a long stay in hospital.
<u>Do you want resuscitation measures in this situation?</u>

○ not wanted	○ probably not wanted	○ indeterminate	○ Probably wanted	○ wanted

Scenario P3.3

The infection should now be treated with antibiotics, otherwise it is possible that you will die. With the antibiotics, there is a high probability that the infection can be treated and that you will be very weak after several weeks in hospital and have little physical strength for everyday and leisure activities.
<u>In this case, do you want antibiotic treatment?</u>

○ not wanted	○ probably not wanted	○ indeterminate	○ Probably wanted	○ wanted

- ○ **I will use the attached copy of my living will as my own living will.**

- ○ **I have further questions about the living will and the study, which is why I would like to be contacted by telephone.**

  telephone number: ____
